# Incidence of seed migration to the chest, abdomen, and pelvis after transperineal interstitial prostate brachytherapy with loose ^125^I seeds

**DOI:** 10.1186/1748-717X-6-130

**Published:** 2011-10-05

**Authors:** Akitomo Sugawara, Jun Nakashima, Etsuo Kunieda, Hirohiko Nagata, Ryuichi Mizuno, Satoshi Seki, Yutaka Shiraishi, Ryuichi Kouta, Mototsugu Oya, Naoyuki Shigematsu

**Affiliations:** 1 Department of Radiology, Keio University School of Medicine, Tokyo, Japan; 2 Department of Urology, Tokyo Medical University, Tokyo, Japan; 3 Department of Radiation Oncology, Tokai University School of Medicine, Isehara, Japan; 4 Department of Urology, Keio University School of Medicine, Tokyo, Japan

**Keywords:** Brachytherapy, ^125^I, Migration, Prostate cancer, Seed

## Abstract

**Background:**

The aim was to determine the incidence of seed migration not only to the chest, but also to the abdomen and pelvis after transperineal interstitial prostate brachytherapy with loose ^125^I seeds.

**Methods:**

We reviewed the records of 267 patients who underwent prostate brachytherapy with loose ^125^I seeds. After seed implantation, orthogonal chest radiographs, an abdominal radiograph, and a pelvic radiograph were undertaken routinely to document the occurrence and sites of seed migration. The incidence of seed migration to the chest, abdomen, and pelvis was calculated. All patients who had seed migration to the abdomen and pelvis subsequently underwent a computed tomography scan to identify the exact location of the migrated seeds. Postimplant dosimetric analysis was undertaken, and dosimetric results were compared between patients with and without seed migration.

**Results:**

A total of 19,236 seeds were implanted in 267 patients. Overall, 91 of 19,236 (0.47%) seeds migrated in 66 of 267 (24.7%) patients. Sixty-nine (0.36%) seeds migrated to the chest in 54 (20.2%) patients. Seven (0.036%) seeds migrated to the abdomen in six (2.2%) patients. Fifteen (0.078%) seeds migrated to the pelvis in 15 (5.6%) patients. Seed migration occurred predominantly within two weeks after seed implantation. None of the 66 patients had symptoms related to the migrated seeds. Postimplant prostate D90 was not significantly different between patients with and without seed migration.

**Conclusion:**

We showed the incidence of seed migration to the chest, abdomen and pelvis. Seed migration did not have a significant effect on postimplant prostate D90.

## Background

Seed migration is a well-recognized event that occurs after transperineal interstitial prostate brachytherapy, and it is observed more often with loose seeds than with linked seeds [1-5].

It is well known that the most frequent site of seed migration is the chest. The American Brachytherapy Society has advised that a chest radiograph should be undertaken at the first follow-up visit to scan the lungs for embolized seeds [6]. Consequently, the incidence of seed migration to the chest has been well reported [1,2,4,5,7-19]. However, documentation of the incidence of seed migration to the abdomen and pelvis is rare. Rare cases of seed migration to a coronary artery, the right ventricle, the liver, the kidneys, Batson's vertebral venous plexus, and the left testicular vein have been reported [20-26]. However, it has never been fully determined whether seed migration to these locations is really rare.

The primary purposes of the present study were to determine the incidence of seed migration not only to the chest, but also to the abdomen and the pelvis at our institution and to identify the exact location of the seeds that had migrated to the abdomen and pelvis with computed tomography (CT). The secondary purpose was to determine the impact of seed migration on postimplant dosimetry.

## Methods

We reviewed the records of 267 patients who underwent transperineal interstitial prostate brachytherapy with loose ^125^I seeds for clinical T1/T2 prostate cancer at our institution. Table 1 details the characteristics of all 267 patients. Two patients (0.75%) received brachytherapy plus external beam radiotherapy (45 Gy in 1.8 Gy fractions). One hundred twenty-three of the 267 (46.1%) patients also underwent neoadjuvant hormonal therapy (NHT), which consisted of luteinizing hormone-releasing hormone agonist and antiandrogens. NHT was generally undertaken in patients with a prostate volume >40 cc or those with pubic arch interference by transrectal ultrasound (TRUS) at the preimplant volume study [27].

**Table 1:** Patient Characteristics (N = 267)

Variable	Value	Range
Age (y)	68.7 ± 0.4	(53-81)
Initial PSA (ng/mL)	7.36 ± 0.20	(4.01-19.88)
Gleason score < 7, n (%)	172 (64.4)	
Gleason score = 7, n (%)	95 (35.6)	
Preimplant prostate volume by TRUS (cc)	23.5 ± 0.4	(9.3-41.0)
Total radioactivity (MBq)	922.3 ± 10.3	(482.9-1300.5)
Number of seeds implanted	72.0 ± 0.7	(40-100)
Number of needles	24.9 ± 0.3	(12-39)

Data are presented as mean ± standard error (range) or number (percent) of patients.

*Abbreviations: *PSA = prostate-specific antigen; TRUS = transrectal ultrasound

One month before seed implantation, a preplan was obtained with TRUS images taken at 5 mm intervals from the base to the apex of the prostate with the patient in the dorsal lithotomy position. The planning target volume included the prostate gland, with a margin of 3 mm anteriorly and laterally and 5 mm in the cranial and caudal directions. No margin was added posteriorly at the rectal interface. Treatment planning used a peripheral or a modified peripheral approach. For the 265 patients who received brachytherapy alone, the prescribed brachytherapy dose was 145 Gy and 160 Gy for the first 163 patients and the subsequent 102 patients, respectively. For the remaining two patients who received brachytherapy plus external beam radiotherapy, the prescribed brachytherapy dose was 110 Gy. TG 43 formalism was used in the preplanning and postimplant dosimetry analyses [28]. All 267 patients were treated with loose ^125^I radioactive seeds with a Mick applicator (Mick Radio-Nuclear Instruments, Bronx, NY). To ensure that no seeds were left in the bladder, postoperative fluoroscopic images were obtained. Prior to discharge, postoperative surveys of voided urine were conducted to detect voided seeds.

Orthogonal chest radiographs, an abdominal radiograph, and a pelvic radiograph were undertaken to document the occurrence and sites of seed migration one day after seed implantation. These follow-up radiographs were undertaken routinely at each outpatient visit. Patients returned to our outpatient clinic two weeks and three months after seed implantation, then at three-month intervals for the first three years and at six-month intervals thereafter. Seed migration to the chest and the abdomen was recorded when one or more seeds were visualized on orthogonal chest radiographs and the anteroposterior (AP) abdominal radiograph, respectively. Seed migration to the pelvis was recorded when one or more seeds were separated from the main seed cluster on an AP pelvic radiograph. However, seeds placed into the bladder and the seminal vesicles or seeds placed inferior to the prostate by mistake were not scored as migrated. Seeds voided in the urine were not scored as migrated. Subsequently, all patients who had seed migration to the abdomen and pelvis underwent a CT scan to identify the exact location of the migrated seeds. The incidence of seed migration to the chest, abdomen, and pelvis was calculated.

Postimplant dosimetric analysis by CT was performed one month after seed implantation. The seed count in the region of the prostate gland was determined on the AP pelvic radiographs obtained two weeks after seed implantation. The postimplant prostate D90 (the dose received by 90% of the volume of the prostate) value was compared between patients with and without seed migration. Statistical analysis was performed with Student's t-test. A p value of <0.05 was considered statistically significant.

## Results

In total, 19,236 seeds were implanted in 267 patients. All 267 patients underwent follow-up radiographs. Median follow-up was 41 months (range, 8.5-76 months).

At one day after seed implantation, follow-up radiographs demonstrated that 41 of the 19,236 (0.21%) seeds migrated in 37 of the 267 (13.9%) patients: three seeds in one patient, two seeds in each of two patients, and a single seed in each of the remaining 34 patients. Fifteen (0.078%) seeds migrated to the chest in 15 (5.6%) patients. One (0.0052%) seed migrated to the abdomen in one (0.37%) patient. Twenty-five (0.13%) seeds migrated to the pelvis in 23 (8.6%) patients.

At two weeks after seed implantation, 85 of the 19,236 (0.44%) seeds migrated in 61 of the 267 (22.8%) patients: seven seeds in one patient, three seeds in each of four patients, two seeds in each of 10 patients, and a single seed in each of the remaining 46 patients. Sixty-one (0.32%) seeds migrated to the chest in 48 (18.0%) patients. Seven (0.036%) seeds migrated to the abdomen in six (2.2%) patients. Seventeen (0.088%) seeds migrated to the pelvis in 16 (6.0%) patients.

At three months after seed implantation, 87 of the 19,236 (0.45%) seeds migrated in 63 of the 267 (23.6%) patients: seven seeds in one patient, three seeds in each of four patients, two seeds in each of 10 patients, and a single seed in each of the remaining 48 patients. Sixty-three (0.33%) seeds migrated to the chest in 50 (18.7%) patients. Seven (0.036%) seeds migrated to the abdomen in six (2.2%) patients. Seventeen (0.088%) seeds migrated to the pelvis in 16 (6.0%) patients.

Although seed migration occurred predominantly within two weeks after seed implantation, eventually, six seeds were found to migrate or relocate to the chest long after seed implantation: four seeds were found to have migrated to the chest at a median of 27 months (range 9.1-16 months), and two seeds were found to have relocated from the pelvis to the chest at 6.1 and 48 months after seed implantation, respectively. In these patients, follow-up radiographs were undertaken routinely at every outpatient visit; however, migration or relocation of these seeds was not found at the previous visit. Meanwhile, no seed relocation from the chest to other sites was observed in the present study.

Eventually, 91 of the 19,236 (0.47%) seeds migrated in 66 of the 267 (24.7%) patients: seven seeds in one patient, three seeds in each of five patients, two seeds in each of nine patients, and a single seed in each of the remaining 51 patients. Sixty-nine (0.36%) seeds migrated to the chest in 54 (20.2%) patients. Seven (0.036%) seeds migrated to the abdomen in six (2.2%) patients. Fifteen (0.078%) seeds migrated to the pelvis in 15 (5.6%) patients. All 66 patients were informed of seed migration; none of these patients had symptoms related to the migrated seeds.

### Seeds that migrated to the abdomen (seven seeds in six patients)

**Figure 1:**
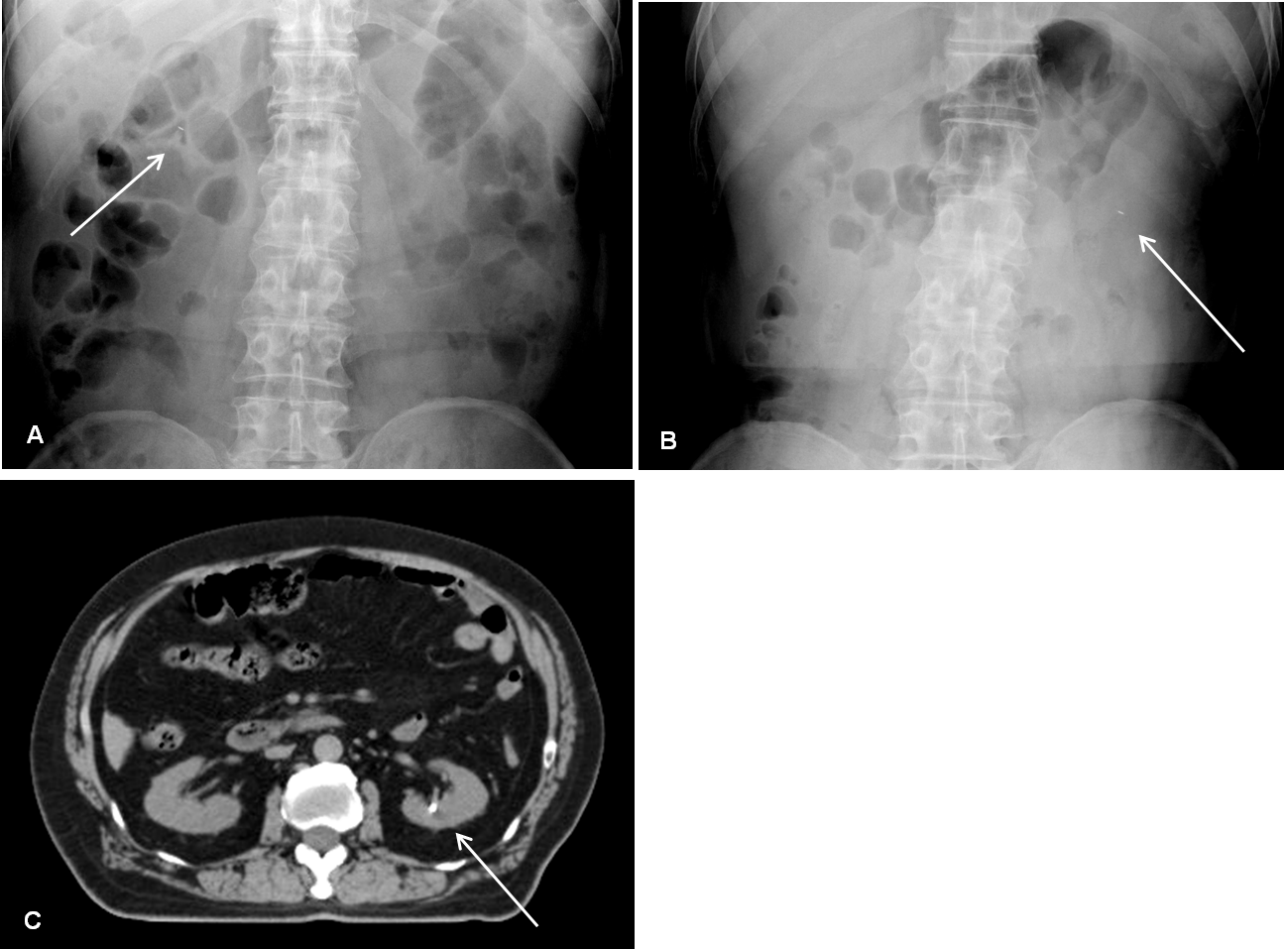
**Case 1: Relocation from the right side of the middle abdomen to the left kidney**. One day after seed implantation, a follow-up abdominal radiograph showed that a seed had migrated to the right side of the middle abdomen (solid arrow) (A). Two weeks later, the seed had relocated from the right side of the middle abdomen to the left side of the middle abdomen (solid arrow) (B). Subsequent computed tomography showed that the seed had migrated to the left kidney (solid arrow) (C).

**Figure 2:**
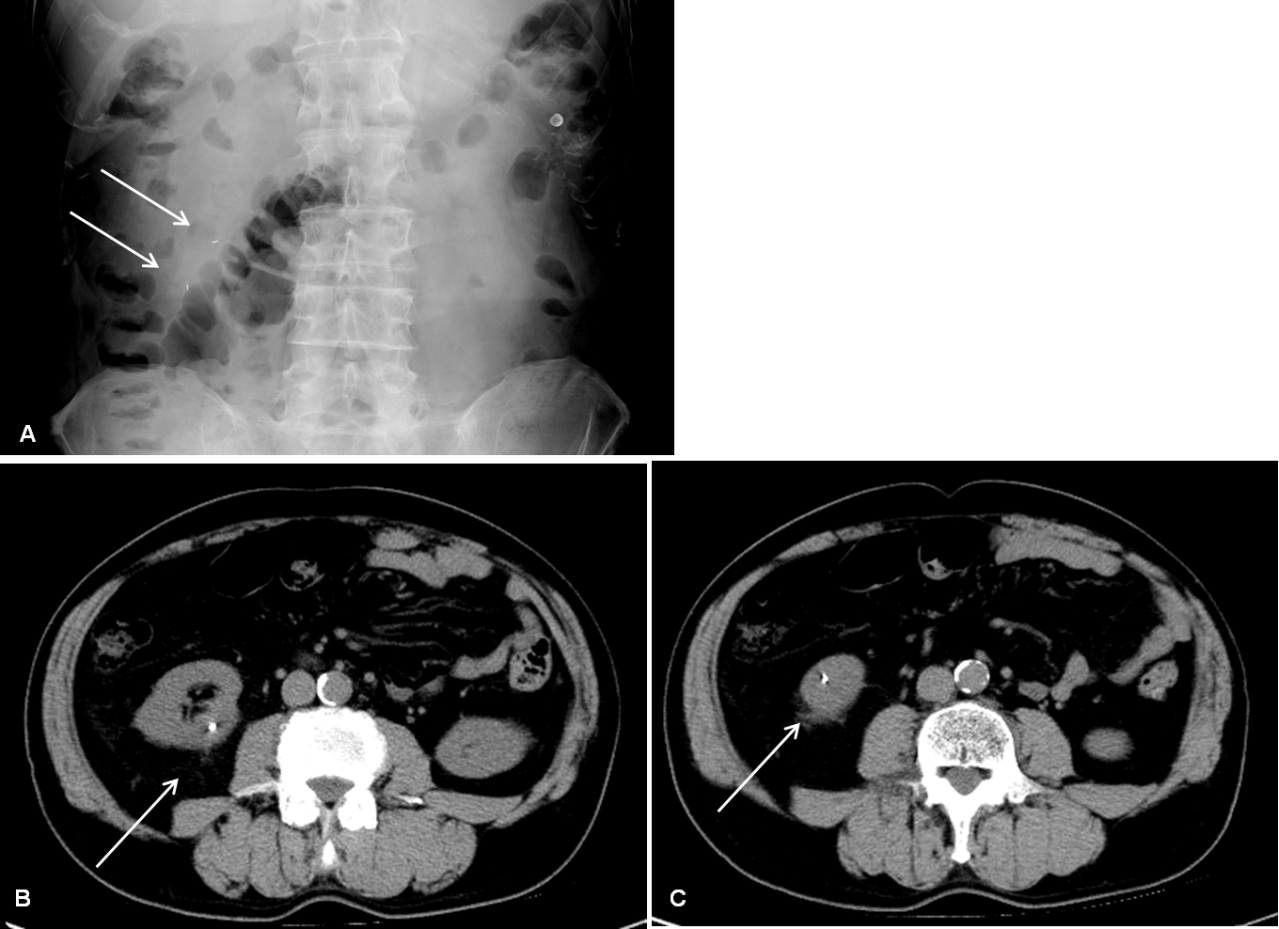
**Case 2: Migration of two seeds to the same right kidney**. Two weeks after seed implantation, a follow-up abdominal radiograph showed that two seeds had migrated to the right side of the middle abdomen (solid arrows) (A). Subsequent computed tomography showed that these two seeds had migrated to the same right kidney (solid arrows) (B,C).

Two of the 19,236 (0.010%) seeds migrated to the liver in two of the 267 (0.75%) patients: a single seed migrated to the liver in each of two patients. Five (0.026%) seeds migrated to the kidneys in four (1.5%) patients: two seeds migrated to the same kidney in one patient, and a single seed migrated to the kidney in each of the remaining three patients. In one patient (*Case 1*), one day after seed implantation, an abdominal radiograph showed that a seed had migrated to the right side of the middle abdomen, which was considered to be separated from the inferior vena cava (IVC) (Figure 1A). However, two weeks after seed implantation, an abdominal radiograph showed that the seed had disappeared from the right side of the middle abdomen, and showed a seed that had migrated to the left side of the middle abdomen (Figure 1B). On pelvic radiographs, there were no changes in number of seeds that had been implanted into the prostate between one day and two weeks after seed implantation. It was concluded that the seed had relocated from the right side of the middle abdomen to the left side of the middle abdomen. A subsequent abdominal CT demonstrated that the seed had migrated to the left kidney (Figure 1C). In another patient (*Case 2*), two weeks after seed implantation, an abdominal radiograph showed that two seeds had migrated to the same right kidney (Figure 2A-C).

### Seeds that migrated to the pelvis (15 seeds in 15 patients)

A single seed migrated to the pelvis in each of 15 patients. Five of the 19,236 (0.026%) seeds migrated to Batson's vertebral venous plexus in five of the 267 (1.9%) patients. Four (0.021%) seeds migrated to the sacral venous plexus in four (1.5%) patients. Two (0.010%) seeds migrated to the iliac veins in two (0.75%) patients. Two (0.010%) seeds migrated to the right ischial bone in two (0.75%) patients. Two (0.010%) seeds migrated to the obturator internus muscles in two (0.75%) patients.

### Seed relocation four years after seed implantation

**Figure 3:**
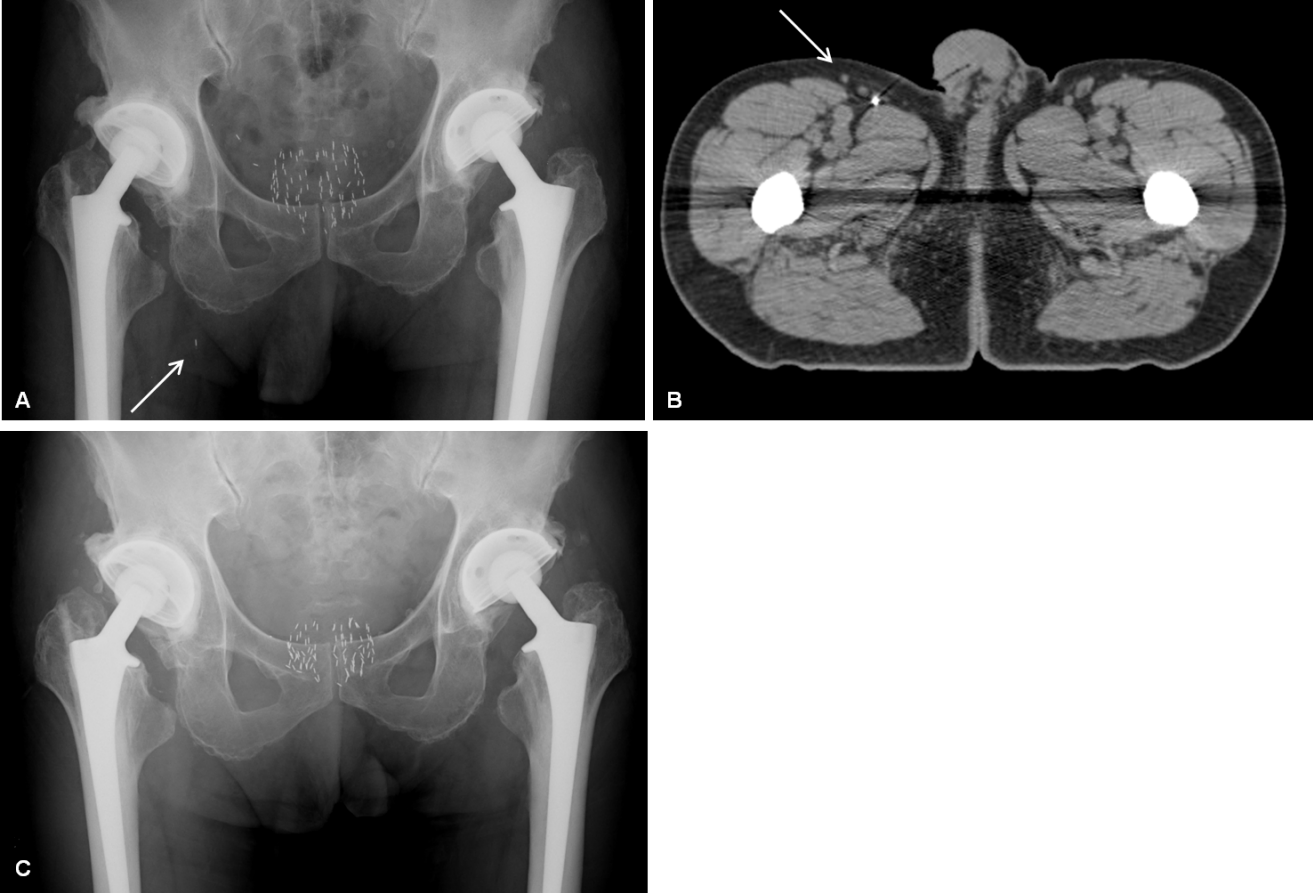
**Case 3: Seed disappearance from the right groin area four years after seed implantation**. One day after seed implantation, a follow-up pelvic radiograph showed that a seed was located inferior to the right side of the pelvis (solid arrow) (A). Subsequent computed tomography showed that the seed had migrated to the right groin area (solid arrow) (B). Four years after seed implantation, a follow-up pelvic radiograph showed that the seed had disappeared from the pelvic area (C).

In one patient (*Case 3*), seed relocation was found four years after seed implantation. A seed had migrated to the right groin area one day after seed implantation (Figure 3A-B). Three years and six months after seed implantation, a pelvic radiograph showed that the seed was in the same location. However, four years after seed implantation, a pelvic radiograph showed that the seed had disappeared from the right groin area (Figure 3C), and a chest radiograph showed a seed that had migrated to the left lung, which was not found at three years and six months after seed implantation. It was concluded that the seed had initially lodged in a branch of the right femoral vein, and then relocated to the left lung through the IVC, long after seed implantation.

### Postimplant dosimetric analysis

In the 265 patients who received brachytherapy alone, the postimplant prostate D90 was 175.0 ± 1.3 Gy (mean ± standard error [SE]). In these 265 patients, the postimplant prostate D90 value was not significantly different between patients with and without seed migration (mean ± SE, 175.1 ± 2.3 Gy vs. 175.0 ± 1.5 Gy, respectively, p = 0.992). In 15 patients who had multiple migrated seeds, the postimplant prostate D90 ranged from 137.2 to 198.5 Gy (mean ± SE, 171.8 ± 5.5 Gy), which was not significantly different from that in patients without seed migration (p = 0.573).

In the remaining two patients who received brachytherapy plus external beam radiotherapy, the postimplant prostate D90 values were 116.4 Gy and 132.6 Gy. These two patients had no seed migration.

## Discussion

### Incidence of seed migration

We found that 0.36% of implanted seeds migrated to the chest in 20% of our patient population, similar to previous reports (Table 2). It has been reported that the incidence of seed migration to the chest can be as high as 55% per patient population and 0.98% per number of implanted seeds (Table 2) [9]. The variability of the incidence of seed migration to the chest among the reported results is considered to be attributed to different types of seeds (linked or loose), different designs of seed placement (intraprostatic or extraprostatic), different timings of follow-up radiographs, and different protocols of follow-up chest radiographs (orthogonal or AP alone) (Table 2) [1-5,9,13].

**Table 2:** Literature Survey of Seed Migration

			Patients with seed migration		Seed migration rate			
						
Study [ref.]	Isotope	Loose or linked	Number	%	Number	%	Location	Timing of radiographs
Kumar *et al. *[11]	^125^I	Linked	0/14	0.0				
Steinfeld *et al. *[17]	^125^I	Loose	1/5	20.0	5/600	0.80	Chest	
Grimm *et al. *[10]	^125^I/^103^Pd	Loose	39/221	17.6			Chest	The day of implant
Nag *et al. *[14]	^103^Pd	Loose	6/30	20.0	7/3213	0.22	Chest	The day of implant or the next follow-up visit (< 1 year)
Nag *et al. *[15]	^103^Pd	Loose	19/107	17.8	32/10612	0.30	Chest	Median of 30 days
Tapen *et al. *[5]	^125^I	Linked*	1/143	0.7			Chest	The day of implant
	^125^I	Loose	1/10	10.0			Chest	The day of implant
	^103^Pd	Loose	15/136	11.0			Chest	The day of implant
	Overall		17/289	5.9			Chest	The day of implant
Merrick *et al. *[13]	^125^I	Linked/loose†	18/84	21.4	24/13467	0.18	Chest	<14 days, or >30 days ††
	^103^Pd	loose	16/72	22.2	29/10338	0.28	Chest	<14 days, or >30 days §
	Overall		34/156	21.8	53/23805	0.22	Chest	<14 days, or >30 days ||
Older *et al. *[16]	^103^Pd	Loose	32/110	29.0			Chest	Median of 3 months
Ankem *et al. *[7]	^125^I/^103^Pd	Loose	21/58	36.2	34/4755	0.72	Chest	Median of 45 days
Eshleman *et al. *[9]	^125^I/^103^Pd	Loose	55/100	55.0	119/12135	0.98	Chest	2-3 month
Al-Qaisieh *et al. *[1]	^125^I	Linked	0/100	0.0			Chest	Median of 53 days
Chauveinc *et al. *[8]	^125^I	Loose	27/170	16.0	32/12179	0.26	Chest	2 months
Kunos *et al. *[12]	^125^I/^103^Pd	Loose	43/120	35.8	68/12524	0.54	Chest	Median of 133 days
	^125^I/^103^Pd	Loose			41/12524	0.33	Pelvis	
Fuller *et al. *[2]	^125^I/^103^Pd	Loose	9/37	24.0	11/5688	0.193	Chest	3-12 months
	^125^I/^103^Pd	Loose	2/37	5.4	2/5688	0.035	Pelvis	
	^125^I/^103^Pd	Linked¶	0/23	0.0	0/4018	0.0	Chest	
	^125^I/^103^Pd	Linked¶	0/23	0.0	0/4018	0.0	Pelvis	
Stone *et al. *[18]	^125^I	Loose	4/146	2.7			Chest	Median of 30 months
	^103^Pd	Loose	0/92	0.0			Chest	
	Overall		4/238	1.7	10/21654	0.046	Chest	
Saibishkumar *et al. *[4]	^125^I	Loose	4/20	20.0	5/2160	0.23	Chest	1 month
	^125^I	Linked	0/20	0.0	0/2236	0.0	Chest	
Sugawara *et al. *[19]	^125^I	Loose			33/11240	0.29	Chest	3 months
	^125^I	Loose			3/11240	0.027	Abdomen	
	^125^I	Loose			10/11240	0.089	Pelvis	
	Overall		35/158	22.2	46/11240	0.41		
Present series	^125^I	Loose	54/267	20	69/19236	0.36	Chest	Median of 41 months
	^125^I	Loose	6/267	2.2	7/19236	0.036	Abdomen	
	^125^I	Loose	15/267	5.6	15/19236	0.078	Pelvis	
	Overall		66/267	25	91/19236	0.47		

*Linked seeds were restricted to needles placed at the periphery of the prostate implant. All seeds placed centrally, close to the urethra, were loose seeds. On average, 8-10 loose seeds were used.

†Of the total ^125^I seeds, 64% were linked seeds. Linked seeds were limited to the region of the capsule/periprostatic region. Loose seeds were utilized in the central aspect of the gland.

††9 patients, within 14 days; 75 patients, more than 30 days.

§11patients within 14 days; 61patients, more than 30 days.

||Of the 20 patients who had postimplant chest radiographs obtained within 14 days of brachytherapy, none had seeds detected in the lungs. Categorizing patients by isotope, for ^125^I, 18/75 (24.0%) and for ^103^Pd, 16/61 (26.2%) with chest radiographs beyond 30 days had seeds found in the lungs.

¶A mixture of linked and free seeds, with a median 74% of needles per case containing seeds in linked, with central and posterior needles typically containing loose seeds.

In contrast, the incidence of seed migration to the abdomen and pelvis has been reported rarely (Table 2). A possible reason is that the American Brachytherapy Society does not specifically recommend follow-up abdominal and pelvic radiographs after seed implantation [6]. Therefore, in most institutions, follow-up abdominal and pelvic radiographs would not be undertaken routinely. However, we found that seed migration to the abdomen and pelvis occurred in 2.2% and 5.6%, respectively, of our patient population. Although the incidence of seed migration to the abdomen and pelvis is lower than that of seed migration to the chest, we would consider it advisable to undertake follow-up abdominal and pelvic radiographs after seed implantation.

### The dynamics of seed migration to the chest

One day, two weeks, and three months after seed implantation, follow-up chest radiographs showed 22%, 88%, and 91%, respectively, of 69 seeds that eventually migrated to the chest. These results mean that 22%, 66%, and 2.9% of these 69 seeds migrated to the chest within one day, between one day and two weeks, and between two weeks and three months after seed implantation, respectively. About 90% of these 69 seeds migrated to the chest within two weeks after seed implantation. These results are similar to a previous report [13]. Merrick et al. have speculated that seed migration to the chest may be most likely to occur between 14 and 28 days after seed implantation [13]. Although we observed that several seeds migrated to the chest more than six months after seed implantation, this finding should be considered exceptional. Therefore, it is suggested that follow-up chest radiographs should be undertaken two weeks, or preferably a few months, after seed implantation to detect most seeds that will migrate to the chest.

### Seed migration to the kidneys and Batson's vertebral plexus is not very rare

The results of the present study show a total of four and five cases of seed migration to the kidneys and Batson's vertebral venous plexus, respectively, at only one institution, which suggests that such cases are not very rare. Meanwhile, in previous studies, a total of only four and four cases of seed migration to the kidneys and Batson's vertebral venous plexus, respectively, have been reported as rare cases, which is in disagreement with our conclusion [22,23,26,29]. The same number or more cases of seed migration to these areas were found in our single study compared with all previous studies. A possible explanation is that, in the present study, orthogonal chest radiographs, an abdominal radiograph, and a pelvic radiograph were undertaken routinely to detect seed migration to the chest, abdomen, and pelvis at several time points after seed implantation. Moreover, in all patients who had seed migration to the abdomen and pelvis, a CT scan was undertaken to identify the exact location of the migrated seeds. Consequently, more cases of seed migration to the kidneys and Batson's vertebral venous plexus were found in the present study. We speculate that some seed migration to the kidneys and Batson's vertebral venous plexus might have gone undetected in other institutions.

### In some cases of seed migration to the kidneys, the mechanism is difficult to explain

Previous investigators have explained seed migration to the kidneys as follows: migrated seeds in venous vessels would enter the systemic circulation through a right-to-left shunt, such as a patent foramen ovale or pulmonary arteriovenous malformation, and then embolize to a branch of the renal arteries [26]. We call this route of seed migration through a right-to-left shunt "a paradoxical route."

The present study has provided some interesting cases of seed migration to the kidneys, the mechanism of which is difficult to explain. In Case 1 of the present study, a seed had migrated to the right side of the middle abdomen, which was considered to be separated from the IVC, and then had relocated to the left kidney (Figure 1A-C). If the seed had initially embolized to an artery of the right side of the middle abdomen, such as a branch of the right renal artery, through a paradoxical route, it is difficult to explain how the seed would have relocated to the left kidney. In Case 2, two seeds had migrated to the same right kidney (Figure 2A-C). The mechanism of seed migration to the kidney through a paradoxical route is too complicated. Therefore, it is highly unlikely that two seeds would happen to migrate to the same right kidney through a paradoxical route by chance, although the possibility cannot be completely excluded. Other possible mechanisms should be proposed to explain how seed migration to the kidneys would have occurred in the two cases mentioned above.

### Other possible mechanisms of seed migration

We assume that seed movement in venous vessels would reflect not only the force of the blood flow but also the force of gravity, and that a seed sometimes would move in venous vessels against the blood flow, because the gravity could overcome the blood flow. The explanation is as follows. Intravascular missile migration following gunshot injury has been reported [30-34]. A missile sometimes moves in a retrograde fashion against the normal blood flow in major venous vessels including the IVC, and sometimes lodges in the renal vein and the hepatic vein [30-34]. It has been postulated that missile migration against the blood flow in venous vessels could occur because of gravity, the patient's position (upright) at the moment of wounding and/or positional changes of the body, the weight and shape of the missile, and possible low flow states [30]. Although the size and the weight of an ^125^I radioactive seed (4.5 mm in length, 0.8 mm in diameter, and about 10 mg in weight per seed) are smaller than those of a bullet, it is considered that a seed could move against the blood flow in major venous vessels because of gravity. The reasons are as follows. The specific gravity of the seed (about 4 g/mL) is much higher than that of blood, and the seed is less water resistant because of its rod-shaped structure. In addition, a seed would usually be located near the wall of major venous vessels due to gravity and the patient's position, and therefore, a seed would be affected by a relatively slow stream of blood near the vascular wall compared with that in the center of major venous vessels. It is known that in major venous vessels, the blood flow is generally laminar, and the distribution of velocity across the tube is parabolic [35]. Therefore, the velocity of blood flow decreases from the center toward the wall of the venous vessel tube [35].

The mechanism of seed migration to various sites could be explained by the assumption that a seed could move in major venous vessels against the blood flow by gravity. In the present cases, seed migration to the kidneys, the liver, and the right groin area (probably a branch of the right femoral vein) (Case 3) would have occurred in venous vessels, partially in a retrograde fashion by gravity, without being passed through a paradoxical route. This explanation does not require the assumption that a patient would have a right-to-left shunt.

### Influence of seed migration on postimplant dosimetry

The postimplant prostate D90 was not significantly different between patients with and without seed migration. Moreover, in 15 patients who had multiple migrated seeds, the postimplant prostate D90 was relatively acceptable, and no supplemental seed implantation was required. These results indicate that seed migration did not have a significant effect on postimplant prostate dosimetry in the present study. Possible reasons are as follows. First, in most patients with seed migration, only one or two seeds had migrated, which would have less effect on the dosimetry of the prostate. Tapen et al. have suggested that the loss of a few seeds may not have a significant effect on dose homogeneity or total dose to the prostate [5]. Second, seed migration would have much less effect on the dosimetry of the prostate than other mechanisms of seed loss, such as seed misplacement to the seminal vesicle or perineum and being voided in the urine postoperatively. Merrick et al. have reported that seed migration to the chest accounted for only 10% of total seed loss from the prostate region, highlighting the importance of other mechanisms of loss [13].

### Seed migration-related sequelae

In the present study, none of the 66 patients had symptoms related to the migrated seeds. Although it has been reported that most patients with seed migration are asymptomatic, there have been a few reports of seed migration-related sequelae, such as a few anecdotal instances of cardiac arrhythmia, myocardial infarction, and radiation pneumonitis [21,36,37]. Therefore, it is important to try to reduce the incidence of seed migration. The use of seeds linked with an absorbable suture material has been associated with a dramatically decreased rate of seed migration, although a potentially higher risk of radiotoxicity to the urethra and rectum has been pointed out [1,3,5,38]. In our study, linked seeds were not administered, because they are not commercially available in our country at present.

## Conclusions

In the present study, we determined the incidence of seed migration not only to the chest, but also to the abdomen and pelvis. Although the incidence of seed migration to the abdomen and pelvis was lower than that of seed migration to the chest, it would be advisable to undertake follow-up abdominal and pelvic radiographs after seed implantation. Seed migration to the chest occurred predominantly within two weeks and, therefore, it is suggested that follow-up chest radiographs should be undertaken two weeks or later after seed implantation. Seed migration to the kidneys and Batson's vertebral venous plexus was not very rare. Seed migration did not significantly affect postimplant prostate D90.

## Competing interests

The authors declare that they have no competing interests.

## Authors' contributions

AS and JN designed the study, collected the data, interpreted the results, performed the statistical analysis, drafted the manuscript, and oversaw the project's completion. EK, HN, RM, SS, YS and RK participated in data acquisition. MO and NS contributed to data analysis. All authors read and approved the manuscript.
